# Intestinal Expression of miR-130b, miR-410b, and miR-98a in Experimental Canine Echinococcosis by Stem-Loop RT-qPCR

**DOI:** 10.3389/fvets.2020.00507

**Published:** 2020-08-26

**Authors:** Ashkan Faridi, Ali Afgar, Seyed Mohammad Mousavi, Saeid Nasibi, Mohammad Ali Mohammadi, Mohammad Farajli Abbasi, Majid Fasihi Harandi

**Affiliations:** ^1^Student Research Committee, Kerman University of Medical Sciences, Kerman, Iran; ^2^Research Center for Hydatid Disease in Iran, Kerman University of Medical Sciences, Kerman, Iran; ^3^Neuroscience Research Center, Institute of Neuropharmacology, Kerman University of Medical Sciences, Kerman, Iran

**Keywords:** *Echinococcus granulosus*, dog, host response, small intestine, miRNA, stem-loop RT-qPCR

## Abstract

*Echinococcus granulosus* is a zoonotic cestode dwelling in the small intestine of canid definitive hosts. Intermediate hosts are a wide range of domestic and wild ungulates. Human infection with the larval stage causes cystic echinococcosis. Understanding the nature and extent of molecular mechanisms involved in host–parasite interactions helps to answer some very basic questions in the biology of cestode parasites with significant implications in the management and control of cystic echinococcosis. Little is known on the miRNAs expression in the intestinal tissues of dogs infected with *E. granulosus*. In the present study, expression of a selected profile of miRNAs was evaluated in experimental canine echinococcosis. MiRNAs were extracted from 20 different parts of small intestinal tract of two sibling 3-months-old mix-breed dogs. Complementary DNA was specifically synthesized using an optimized stem-loop system. Intestinal expression of four miRNAs (cfa-let7g, cfa-miR-98, cfamiR-410, cfa-miR-130b) was evaluated using RT-qPCR. The results of the study indicate a significant difference between test and control dogs in cfamiR-130b, cfa-miR-98, and cfa-miR-410 (*P* ≤ 0.05); however, there was no significant difference for cfa-let7g. The most upregulated miRNAs were cfamiR-130b and cfa-miR-98. An increasing trend for cfa-let7g and a declining trend for cfa-miR-98, cfa-miR-410, and cfamiR-130b were found toward the distal segments of the small intestine. Our study revealed that cfa-miR-98, cfa-miR-410, and cfamiR-130b are involved in the definitive host response in canine echinococcosis. The study provides new information on the molecular basis of interactions between *E. granulosus* and dogs in terms of miRNA expression and showed that *E. granulosus* infection could increase the expression of some pro-inflammatory miRNAs at the cellular level in the definitive host.

## Introduction

Cystic echinococcosis caused by the small cyclophyllidean tapeworm *Echinococcus granulosus* is among the most important cyclozoonoses of the world (1). In the life cycle, the carnivores as definitive hosts carry the adult tapeworms in the small intestine, and a wide range of mammals including humans harbor the metacestode (hydatid cyst) in their internal organs, mainly liver and lung, as the intermediate host. In the endemic areas, domestic dog-sheep rather than sylvatic wolf-deer life cycle is the most dominant cycle for *E. granulosus* (2). Global cases have been estimated at 2–3 million (3), and according to the WHO, the annual cumulative economic losses in humans and livestock are estimated to be US$3 billion in the world^1^.

Dogs infected with *E. granulosus* hardly experience any clinical sign, and due to their close relationship with humans (4), they are assumed to be a potential risk for disease transmission to a human being. Recently, impressive research efforts have been directed toward understanding the interactions between a host and a parasite and unlocking the molecular mechanisms involved in the host responses to helminthic infections (5). Little is known on the extent to which the host immunity is involved in the host–parasite system (6). Cytokines, secreted by various cells in response to different stimuli, are one of the key communication tools between cells and are critical in the immune system regulation. Dominance of type II cytokines (especially IL-4, IL-10) with a decrease in the secretion of type I cytokines (TNF) is the main characteristic of helminth infections (7). The complexity in the function of cytokines in experimental infections is an important challenge to understand the nature of the polarization toward type I or II responses. Several factors may interfere in the expression of cytokine-encoding genes including miRNA (8–10).

Micro-ribonucleic acids (miRNAs) are short (18–25 nt), non-coding evolutionarily protected ribonucleic acids, first discovered in 1990s in the free-living helminth *Caenorhabditis elegans* (11). MiRNAs have been found in plants, animals, and some viruses whose role are to fine-tune gene expression at the post-transcriptional level by mRNA degradation or inhibition of translation (12). These molecular structures are involved in the control of cellular physiological and pathological processes (13). In animals, gene expression regulation is a complex process, such that one miRNA may have hundreds of different target mRNAs, and one target mRNA molecule is regulated by multiple miRNAs (14).

Little information exists on intestinal tissue expression of miRNAs in canine echinococcosis. Most of miRNA studies focused either on different stages of the parasites or the intermediate hosts (15). Our knowledge on the dynamics of miRNA expression in the definitive host is very poor. In a study on Kazakh sheep that is partially resistant to infection with *Echinococcus granulosus*, six miRNAs were shown to be upregulated in ovine hepatic tissues including miR-21-3p, miR-542-5p, miR-671, miR-134-5p, miR-26b, and miR-27a (16). Studies on other parasitic organisms showed downregulation of let-7 and miR-98 of intestinal epithelial cells in response to *Cryptosporidium parvum* infection *in vitro* (17). Another study on intestinal miRNAs of male BALB/c mice indicates that miRNA-155 overexpression associated with dysregulation in the intestinal epithelial apical junctional complex in severe acute pancreatitis (18).

A tissue atlas for dog miRNAs has been developed by Koenig et al. (19) in which 60 out of 106 tissue miRNAs were found highly expressed in a single organ compared to other organs of non-sibling beagle dogs (19).

More data are available on differential miRNA expression in the helminth parasites of dogs including *E. granulosus*. MiRNA profiles at various development stages of *E. granulosus* revealed the involvement of miRNAs in the growth and development of the parasite (20). In the absence of miRNA data from dogs infected with *E. granulosus*, we cannot understand the exact roles of miRNA in host–parasite interactions. The purpose of the present study was to determine the expression level of a selected profile of miRNAs in dog intestinal tissues experimentally infected with *E. granulosus*. In this study, we designed and optimized a stem-loop RT-qPCR for specific synthesis and amplification of cDNA.

## Materials and Methods

### Ethical Statement

This study was performed according to the animal guidelines established by the Kerman University of Medical Sciences Research Ethics Review Committee for the use of non-human animals in research, ethical approval code IR.KMU.REC.1398.084.

### Experiment Infection

Two mix-breed, 3-months-old, sibling, male dogs were provided by a local vendor. The animals were housed in two separate, 6-m^2^ well-ventilated washable rooms under standard conditions. After adapting to the new environment for 5 days and DHLPP vaccination, the animals were treated with 10 mg/kg Praziquantel forte (Fenbendazole 150 mg, Pyrantel Pamoate 144 mg, and Praziquantel 50 mg). Stool specimens were regularly checked for the presence of parasite eggs, and absence of infection was confirmed. The animals received daily chicken diet plus multivitamin and freshwater *ad libitum*. One dog was randomly selected and experimentally infected with sheep liver hydatid cysts. The viability of the cysts protoscoleces was determined as ≥80% using 0.1% eosin exclusion test. The dogs were taken for walking 1 h a day. From the day dogs arrived in the facility until the end of the study, every single day, the feces were collected on a daily basis by trained personnel. A portion of each fecal material was used for microscopic examination under strict aseptic conditions, and all the rest of the fecal materials were buried according to safe disposal instructions. In addition, the floor was burned with a flamethrower under the supervision of the first author. All surfaces of the housings were washed using detergents and dried by the flamethrower.

Fifty-four days post-infection, when the stool exam showed the presence of *E. granulosus* eggs, the animals were euthanized. We chose to euthanize the dogs 54 days post-infection purposefully to study a well-established infection in the definitive host in terms of immunity and the development of molecular processes in host–parasite interactions. Briefly the procedure was as follows: first, the pre-euthanasia drug (xylazine-ketamine in ratio 1/5, 1 ml/10 kg) was administered intramuscularly, followed by intravenous lethal dose of sodium pentobarbital (21). After dissection, the small intestine was divided into 20 segments, including duodenum and ileum as one segment each, and the jejunum was dissected in 18 segments of 20 cm long each. In the test dog, the worm burden was estimated for each segment. An intestinal piece of tissue was sampled from the center of each segment and preserved in liquid nitrogen until use.

### RNA Isolation and cDNA Synthesis

MiRNA isolation was performed using Trizol RNA extraction protocol with some modifications to increase miRNA yield; briefly, 30 mg intestine tissue was homogenized in 600 ml of cold Trizol, then the lysate was pipetted up and down for about 10 min; 200 μl chloroform was added, pipetted several times, and vortexed, and the sample was placed on ice for about 5 min and centrifuged at 12,000 g for 20 min at 4°C; the aqueous supernatant was carefully transferred to a clean tube, and 100 μl chloroform was added, centrifuged at 12,000 g for 20 min at 4°C; and the aqueous supernatant was transferred to a fresh tube. Afterward, sodium acetate (3 M, pH 5) was added at the proportion of one-fifth to the supernatant, and an equal volume of cold isopropanol was added; the tube was stored in −20°C overnight, centrifuged at 12,000 g for 60 min at 4°C, and the pellet was resuspended in 1 ml of 75% ethanol, centrifuged at 12,000 g for 20 min at 4°C; the RNA pellet was left to dry at room temperature, and finally, RNA was resuspended in 50 μl RNase free water. RNA was stored at −80°C until subsequent use. The quantity and purity of the purified RNA were measured using 260/280 and 260/230 ratios with a NanoDrop Spectrophotometer (ND 1000), and the integrity was checked with 1.5% agarose gel electrophoresis (Supplementary Figure 1). An equal amount of all isolated RNA was used to synthesize cDNA by using a stem loop specifically designed for each miRNA separately (see below). Stem-loop opening was performed in a thermocycler at 65°C for 10 min by using 1.5 μl of 100 pm of stem loop, 1,000 ng total RNA, and RNase free water to a final volume of 10.5 μl and then placed on ice for 5 min; afterward, 1 μl dNTP (10 mM each), 1 μl M-MLV (200 U/μl) reverse transcriptase, 4 μl 5 × first-strand buffer [250 mM Tris-HCl (pH 8.3 at 25°C), 375 mM KCl, 15 mM MgCl_2_, 50 mM DTT], and 0.5 μl RNasin (400 U/μl) were added and incubated for 60 min at 44°C. Finally, the reaction was terminated at 95°C for 2 min.

### miRNA Selection

To evaluate cytokine responses against infection with *E. granulosus*, we compared TNF, IL4, IL10, and heat shock protein 70 (HSP70) genes between test and control dogs. These cytokines were produced by cells in response to stressful conditions due to the exposure to different pathogens like helminth parasites. First, Target scan (http://www.targetscan.org/vert_72/) was searched by selecting the species as *Canis familiaris* and the above-mentioned genes to find highly conserved miRNAs between dogs and humans. Then the highest scores were selected based on the scores obtained from predicted consequential pairing of the target region and miRNA, site type, context score, weighted context score, and conserved branch length. The miRNAs were also crosschecked in MiRbase (http://www.mirbase.org/). Afterward, to find the target genes, the selected miRNAs were searched in MirDB (http://mirdb.org/). Finally, by using miRWALK (http://zmf.umm.uni-heidelberg.de/apps/zmf/mirwalk/predictedmirnagene.html), best miRNAs for targeting the genes of interest were selected based on the prediction by DIANA-mT, miRanda, miRDB, miRWalk, RNAhybrid, PICTAR4, PICTAR5, PITA, RNA22, and TargetScan within miRWalk.

### Stem-Loop Design and Validation

**Figure 1:**
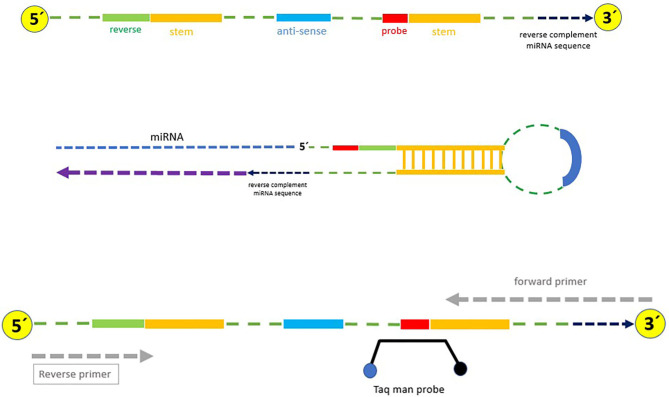
Stem-loop structure specifically designed for cDNA synthesis in this study.

Stem-loop design was carried out based on a robust, specific, and cost-effective technique described by Chen et al. (22) with some modifications (Figure 1 and Table 1). Briefly, (1) the reverse and probe primers were universal; (2) the temperature of the stem loop was reduced for increasing the efficiency of the PCR amplification; (3) only one structure with no additional structures was produced at the temperature in which the cDNA was synthesized (42–45°C); this increased the efficiency and quality of cDNA synthesis as well as the PCR (Supplementary Figure 2A); (4) the stem loop can specifically detect every target miRNA because six nucleotides complementary to the 3′UTR miRNA region were attached to the 3′ end of each stem loop; (5) mature miRNAs were used as forward primers, and the primers were checked by Gene Runner 6.0.28 for secondary structures; and (6) to avoid unwanted secondary structures, the whole amplicon was analyzed by OligoAnalyzer 3.1 (https://www.idtdna.com/pages/tools/oligoanalyzer) (Supplementary Figure 2B). Primer-BLAST was performed for the forward and reverse primers.

**Table 1:** Stem loop used in this study for specific synthesis of cDNA (Green: universal reverse sequence; Yellow: Taq man probe sequence; Blue: 6-nt sequence spacer).

**Stem loop**	**Sequence 5 → 3**
cfa-let7g	GTATGCTGCTACCTCGGACCCTGCTTAGTGCCATGCCTGCCATCGAGCAGCATACAACTGT
cfa-miR-98	GTATGCTGCTACCTCGGACCCTGCTTAGTGCCATGCCTGCCATCGAGCAGCATACTGAGGT
cfamiR-410	GTATGCTGCTACCTCGGACCCTGCTTAGTGCCATGCCTGCCATCGAGCAGCATACAATATA
cfa-miR-130b	GTATGCTGCTACCTCGGACCCTGCTTAGTGCCATGCCTGCCATCGAGCAGCATACCAGTGC
U6	GTATGCTGCTACCTCGGACCCTGCTTAGTGCCATGCCTGCCATCGAGCAGCATACaaaaatatgg
5s	GTATGCTGCTACCTCGGACCCTGCTTAGTGCCATGCCTGCCATCGAGCAGCATACAGCCTA

For validation and determining the limit of detection (LOD), we used the protocol provided by Qiagen (www.qiagen.com). The number of standard molecules of miRNA was quantified using the following formula: {(X g/ml RNA/[RNA Length in nucleotides × 230]) × 6.022 × 1,023 = Y molecule/μl}. Then serial dilutions were prepared for five reactions: 100, 50, 25, 10, and 5. Real-time PCR experiments were performed for each dilution in triplicate. All triplicate reactions were positive for all serial dilutions. The LOD level for miRNA was obtained at 5 copies/reaction.

### Real-Time PCR

Table 1 shows the primer sequences and the expected PCR products for cfa-let7g, cfa-miR-98, cfa-miR-410, cfa-miR-130b, and U6 along with 5S as reference genes. Quantitative RT-PCR of target cDNA was conducted on a Rotor-Gene 6000 (Qiagen, Germany) sequence detection system. The PCR for miRNA detection was carried out in 12.5-μl reaction volumes and included 6.5 μl Probe qPCR master mix, 250 nmol each of forward and reverse primers, 0.5 μl Taq man probe (5′ FAM fluorescent reporter dye and 3′ TAMRA fluorescent quencher dye), and 2 μl cDNA and RNase free water (Tables 2, 3). Two-step PCR conditions were used as follows: 95°C for 3 min, 40 cycles of 95°C for 6 s, and 60°C for 40 s. Two negative controls were used in all sets of reactions, including water as no template control and RT control containing RNA that was not reverse transcribed. For each gene, PCRs were performed in triplicate. PCR results were optimized to the levels of reference genes. DCt was calculated using the following formula: [ΔCT = CT (target) – CT (reference)]. The gene expression level was determined by the 2^−ΔCt^ method. Fold increase (FI) was calculated using the comparative threshold method as 2^−ΔΔCT^. The efficiency of real-time PCR was calculated as 98% using the LinReg software (Supplementary Data File).

**Table 2:** Forward oligonucleotide, universal reverse, and Taq man probe sequence used in this study.

**Forward/Reverse**	**Sequence 5 → 3**	**PCR product (bp)**	**Description**
CFA-LET7G	5GGCTGAGGTAGTAGTTTGTACAGTT3	100	IL10, IL13-TH2
CFA-MIR-98	GGCAACAATACAACTTACTACCTCA	100	HSP 70
CFA MIR-410	GGGACAGGCCATCTGTGTTATATT	100	IL4-TH2
CFAMIR-130B	CATGCCCTTTCATCATTGCACTG	100	TNF-TH1
U6	GCAAGGATGACACGCAAATTCG	100	Reference gene
5S	AATACCGGGTGCTGTAGGCT	100	Reference gene
Universal reverse	GCTGCTACCTCGGACCCT		Reverse
TAQ man probe	AGTGCCATGCCTGCCATCGAGC		Probe

**Table 3:** Genomic location and predicted target of miRNAs in *Canis familiaris*.

**miRNAs**	**Accession number**	**Genome context**	**Predicted gene target sites**
cfa-let7g	(MIMAT0006637)	chr20: 37501074–37501152	483
cfa-miR-98	(MIMAT0006756)	chrX: 45253693–45253772	480
cfa-miR-410	(MIMAT0006728)	chr8: 69294377–69294432	639
cfa-miR-130b	(MIMAT0006659)	chr26: 30924432–30924491	470

### Statistical Analyses

By using the SPSS 22 software, Mann–Whitney test was used to compare the mean of gene expression data, and *P*-values ≤ 0.05 were considered significant. Pearson's correlation coefficient was used in order to determine the association between worm burden and gene expression. The data are given as mean ± standard deviations (SD).

## Results

**Figure 2:**
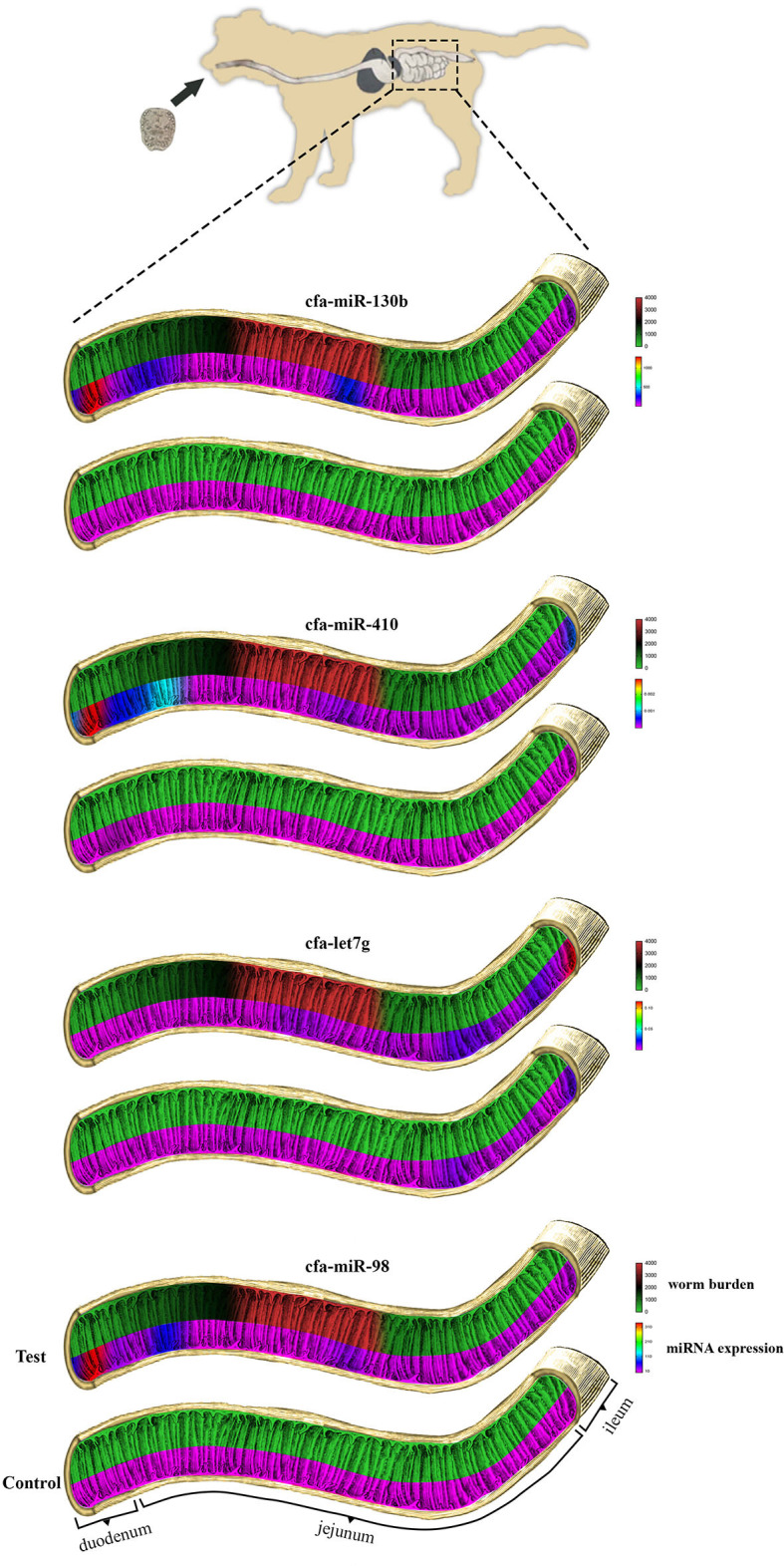
Comparisons of miRNA expression and the number of *E. granulosus* worms recovered in each of the 20 intestinal segments of experimental test and control dogs. From top to bottom: cfa-miR-130b, cfa-miR-410, cfa-let7g, and cfa-miR-98.

**Figure 3:**
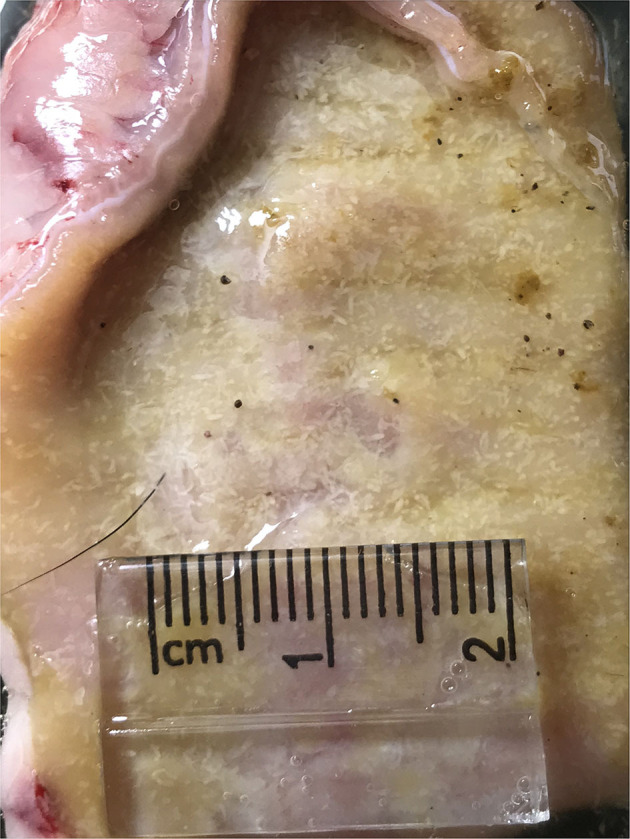
Heavy infection of *Echinococcus granulosus* (white) attached to the small intestine of an infected dog.

As shown in Figure 2, the maximum number of worms attached to the intestinal wall was detected in median segments (#8–12) of the jejunum with more than 2,000 adult worms per segment (Figure 3), whereas the lower burden of infection was found in the proximal and distal segments so that no worm was found at the last segment of the jejunum as well as the ileum. There were no worms in the control dog.

The average quantity of extracted total RNA was found as 4064.7 and 3990.25 ng/μl in the control and infected dog, respectively. The ratio of nucleic acid to protein (according to 260/280 nm absorption) was >2.1, and the ratio of nucleic acid to phenolate ion, thiocyanates, and other organic compound contamination (according to 260/230 nm absorption) was above 1.8. 18S and 28S ribosomal RNAs were seen with 1.5% gel electrophoresis (data not shown).

**Figure 4:**
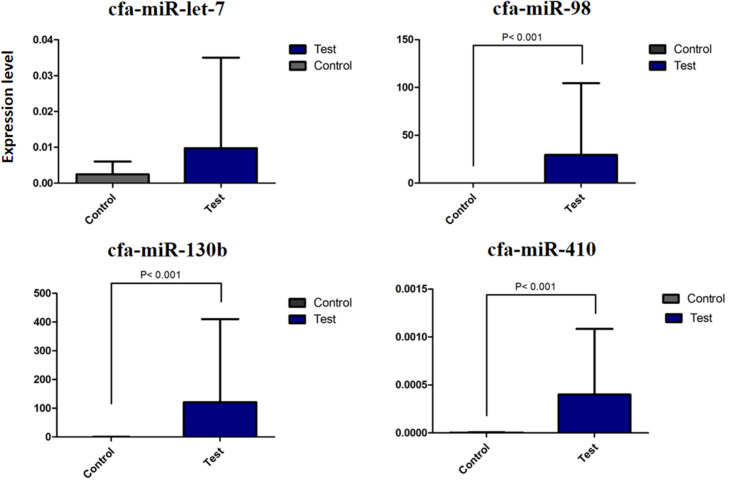
Differential expression of miRNA cfa-let7g, cfa-miR-98, cfa-miR-410, and cfa-miR-130b between control and test dogs in the experimental infection with *E. granulosus*.

The summary of the overall fold change expression between control and test dogs on the miRNAs, cfa-let7g, cfa-miR-98, cfa-miR-410, and cfamiR-130b are presented in Supplementary Figure 3. All the miRNAs demonstrated increased expression in the test in comparison to the control dog. Statistical analysis showed significant differences in cfamiR-130b, cfa-miR-98, and cfa-miR-410 (*P* ≤ 0.05). No significant difference was found for cfa-let7g. The most upregulated miRNAs were cfamiR-130b and cfa-miR-98. Figure 4 demonstrated the regional expression of miRNAs in the small intestine of the test and control dogs.

No special pattern was found in the expression of miRNAs on different segments of the small intestine; however, an increasing trend for cfa-let7g and a declining trend for cfa-miR-98, cfa-miR-410, and cfamiR-130b were found toward the distal segments of the small intestine (Figure 2). A statistically significant correlation between regional expression and worm burden was observed for cfa-miR-98 (*r* = 0.700), cfa-miR-410 (*r* = 0.667), and cfamiR-130b (*r* = 0.722) (*P* ≤ 0.05).

## Discussion

The present study investigated the effects of *E. granulosus* infection on the expression of a selected profile of dog miRNAs potentially involved in the host intestinal immune/inflammatory responses. Our results indicate that experimental infection of *E. granulosus* in dogs resulted in the significant increase in the expression in the miRNAs. Little is known about the status of intestinal miRNAs in dogs infected by *E. granulosus*. Understanding the nature and extent of molecular mechanisms involved in host–parasite interactions helps to answer some very basic questions in the biology and development of cestode parasites with significant implications in the management and control of hydatid disease.

An enormous growth of interest in miRNA research mainly as a diagnostic biomarker of cancer has been seen in recent years (23). For instance, overexpression of miR-122 in colorectal cancer with liver metastasis has been assumed as a potential diagnostic and prognostic biomarker. In *E. granulosus*, increasing significance of miRNA research focuses on the biology and development of the parasite (15, 24). Friedländer et al. (25), by using miRDeep and deep sequencing data, have found that mature miR cfa-miR-130b, cfa-miR-410, cfa-miR-98, and cfa-miR-let-7 are present in the tissue of a normal dog.

The first research question investigated the effect of canine echinococcosis on the expression of cfa-miR-130b. This miRNA is involved in numerous biologic processes including mesenchymal differentiation, adipogenesis suppression (26), TNF receptor superfamily, immune cell function, and oxygen homeostasis (27). As mentioned earlier, very little data are available on the status of miRNA expressions in dog intestinal tissues in disease conditions. A study on gastric cancer revealed that miR-130b regulates the tumor suppressor RUNX3 in gastric cell lines and was identified as the highest candidate for RUNX3 binding miRNA, which leads to improved viability of the cells, decreased cell death, and reduced expression of Bim in TGF-b mediated apoptosis (28). However, in another study on colorectal cancer, upregulation of miRNA-130b was assumed as a tumor developer and associated with poor prognosis in human CRC-derived cell lines (29).

A couple of studies have been conducted on miR-130b expression in extra-intestinal tissues of dogs. In canine multicentric lymphoma, miR-130b had upregulation and was found correlated with both B and T cell lymphoma (30). In canine uveal melanoma, miR-130b expression was found upregulated in metastasizing tumors, and it was suggested as a potential biomarker to identify the metastasizing disease (31). A study on mice obesity-related inflammation revealed that after TNFa stimulation, miR-130 was overexpressed, and this was associated with adipocyte dysfunction (32). In a study to determine the miR-130b expression in osteosarcoma, significant miR-130b upregulation was demonstrated in osteosarcoma tissues compared to the normal adjacent tissues. The miRNA was shown to play a crucial role in the progression of osteosarcoma in patients by targeting PPAR_γ_ (33).

Here we demonstrated that cfa-miR-130b gene expression is highly upregulated in the intestinal cells of dogs infected experimentally with *E. granulosus*, and as it is indicated in several studies, miR-130b could be potentially involved in the pathogenesis and inflammatory responses in the definitive hosts.

We showed that cfa-miR-410 gene expression was upregulated in the intestinal cells of dogs infected experimentally with *E. granulosus*. In an avian-like H5N1 canine influenza virus model, cfa-miR-410 expression was decreased in the lung and tracheal tissues (34). Several studies on miR-410 suggested probable links with immune responses. It has been referred to as a tumor suppressor in pancreatic cancer (35) and as an enhancer of tumor invasion in liver and colorectal malignancies (36). A study on systemic lupus erythematosus showed that miR-410 downregulates IL6 and IL10 expression, and its overexpression significantly reduced the expression levels of TGF-b1 in SV40MES13 cells (37). In liver and colorectal tumors, upregulated miR-410 increased the tumor cell growth by silencing FHL1 tissues/cell lines (36). Another study on human colorectal cancer cells showed that miR-410 may function as an oncogenic miRNA by repressing the basal level of apoptosis (38).

The most studied miRNA family in mammals is let-7 (39); however, data on the expression profiles of this family in dog intestinal tissues are exceedingly poor. The let-7 (lethal-7) miRNA family consists of 9–13 members including mir-98 in mammals (39–41), and it is known as a tumor suppressor (42) and regulates development in some eukaryotic organisms, e.g., *Caenorhabditis elegans* (43). However, in mammalian cells, the specific role of let-7 family members in development has not yet been fully understood (41).

In ulcerative colitis and Crohn's disease, miR-98 and let-7e were found upregulated, and they were suggested as new potential diagnostic biomarkers (44). In two *in vitro* studies on host cholangiocytes and epithelial cells, Hu et al. (17, 45) stimulated cells with LPS/*C. parvum*; consequently, the level of miR-98 and let-7 expression was decreased.

However, it should be noted that the results of *in vivo* studies could hardly be compared with those of *in vitro* studies. Moreover, host interactions in bacterial and protozoan infections have basic differences with intestinal helminth infections. As demonstrated by Hu et al. miR-98 and let-7 regulate the expression of the untranslated region of the Suppressor of Cytokine Signaling 1 and 4 (SOCS1 and SOCS4) that play an important role in host cell response to infection. SOCS gene negatively regulates cytokine signaling (46) and prevents the activation of the JAK-STAT pathway; thus, targeting SOCS leads to the activation of the JAK-STAT pathway (46). JAK-STAT, in turn, induces the expression of genes involved in survival, division of immune cells, activation, and recruitment cell division and stimulates inflammation. As a result, STAT1 activates the expression of multiple pro-inflammatory genes, and this could be used as a potential therapy for inflammatory disorders (47).

Some limitations of the present study need to be acknowledged. Finding numerous same gender sibling dogs was very difficult in terms of ethical, practical, and financial considerations. Regrettably, our knowledge on the host–parasite interactions in canine echinococcosis is extremely poor, partly due to the nature of experimental studies on dogs limiting the research team capabilities to use a satisfactory number of dogs in the experiments. However, the sample size for comparing miRNA expression among 40 intestinal segments was sufficient and statistically suggestive. The study attempted to compare the expression of a selected profile of miRNAs involved in host response in canine echinococcosis; however, it is clear that lots of other miRNAs are potentially involved in this phenomenon, and each miRNA is capable of controlling several target mRNAs along diverse signaling pathways that, in turn, add a further level of complexity in the dynamics of infection. Therefore, more in-depth studies are required to clarify the role of miRNAs in dog–*Echinococcus* interactions.

Very little is known about the nature of *E. granulosus* infection in dogs at the miRNA level, partly due to some ethical and safety issues inherent in such troublesome and awkward studies. Our study provides first-hand evidence toward understanding of the complex mechanisms involved in the dog response to *E. granulosus* infection. The findings of this study have a number of important implications. Here we designed and optimized a stem loop that, to the best of our knowledge, could amplify almost every miRNA according to similar specific 6-nt attached to the 3′ end of the stem-loop structure. This approach could be used in other miRNA studies, replacing commercial miRNA detection kits. The empirical findings in this study provide new information on the interaction between the host and the parasite in terms of miRNA expression and showed that *E. granulosus* infection could increase the expression of some pro-inflammatory miRNAs at the cellular level in the definitive host. Nevertheless, miRNA research in canine echinococcosis is only beginning to come into understanding the susceptibility of dogs to such diseases.

## Data Availability Statement

The authors acknowledge that the data presented in this study must be deposited and made publicly available in an acceptable repository, prior to publication. Frontiers cannot accept a article that does not adhere to our open data policies.

## Ethics Statement

For using non-human animals in the study, the project was reviewed and approved by the Research Ethics Review Committee of Kerman University of Medical Sciences, approval ID IR.KMU.REC.1398.084.

## Author Contributions

AF, AA, and MFH: conceptualization, study design, data validation, and writing–original draft preparation. AF, AA, and MM: data curation. AF, AA, SM, and MFH: data analysis. MFH: funding acquisition. AF, SM, SN, MM, and MFA: laboratory experiments. AF, AA, SM, SN, MM, MFA, and MFH: revising and final approval of the manuscript. All authors contributed to the article and approved the submitted version.

## Conflict of Interest

The authors declare that the research was conducted in the absence of any commercial or financial relationships that could be construed as a potential conflict of interest.

## Footnotes

^1^*Echinococcosis*. Available online at: https://www.who.int/news-room/fact-sheets/detail/echinococcosis (accessed May 1, 2020).
