# Supplementary material for: Intestinal Expression of miR-130b, miR-410b, and miR-98a in Experimental Canine Echinococcosis by Stem-Loop RT-qPCR
Source: Front Vet Sci. 2020 Aug 26;7:507. doi: 10.3389/fvets.2020.00507 (PMC7480022; doi:10.3389/fvets.2020.00507)
Supplement: Supplementary file 1 [file Data_Sheet_1.docx]

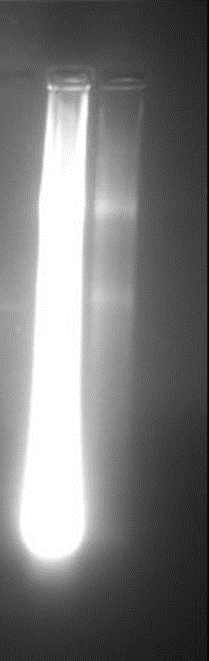


**Supplementary Figure 1.** The integrity of total RNA on 1% agarose gel electrophoresis.

A


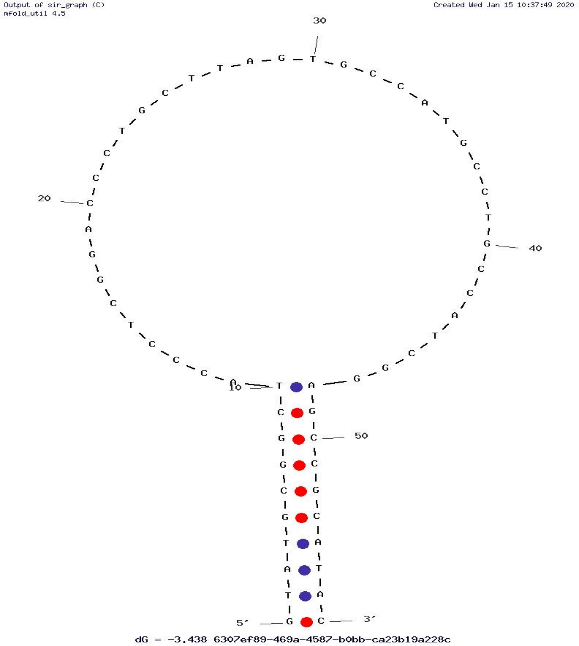


B


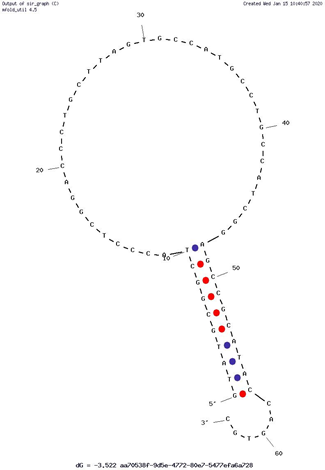


**Supplementary Figure 2.** RT specific stem-loop primer before (A) and after (B) adding six nucleotides complimentary to the 3’UTR of miRNA.

**Supplementary Figure 3.** Overall fold change expression between control and test dogs for miRNA cfa-let7g, cfa-miR-98, cfa-miR-410 and cfamiR-130b.
